# Ecotoxicology in tropical regions

**DOI:** 10.1007/s11356-018-1887-4

**Published:** 2018-04-24

**Authors:** Jonas S. Gunnarsson, Luisa E. Castillo

**Affiliations:** 1 Department of Ecology, Environment and Plant Sciences (DEEP), Stockholm University (SU), Stockholm, Sweden; 2 Regional Institute for Studies on Toxic Substances (IRET), National University (UNA), Heredia, Costa Rica

The present special volume “Ecotoxicology in Tropical Regions” contains 19 research articles that were selected from presentations at two conference sessions at the SETAC Europe 24th and 25th meetings in Basel 2014 and in Barcelona 2015. The papers address major ecotoxicological issues in several tropical and sub-tropical countries including Brazil, Mexico, Costa Rica, Columbia, Nicaragua, Costa Rica, Ethiopia, Thailand, Vietnam, and Iran.

In 2017, more than 16,000 scientists from 184 countries published a “Warning to humanity letter” (Ripple et al. 2017). The letter issued a warning that humans are on an accelerating collision course with our environment, putting the future of humankind and our planet at risk. The authors highlight a series of major threats to Earth, including environmental pollution (industrial pollutants, pesticides, fertilizers, pharmaceuticals, oil, radioactive isotopes), green-house gas emissions leading to temperature increase and climate change, spreading of marine dead zones due to eutrophication and lack of oxygen in the bottom water, deforestation and habitat destruction, species’ extinction and biodiversity loss, overfishing and depletion of freshwater resources. The authors stress that by failing to limit world’s population growth, estimated to reach 10 billions by 2050, and failing to protect natural habitats and to limit human consumption and destruction of our natural resources, we are putting our whole planet and the future of humankind in peril.

More than 90% of the predicted additional two billion people on Earth by 2050 are expected to be born in developing countries in Asia and Africa and most of them in tropical and sub-tropical areas (UN 2017). These countries will thus be exposed to both ongoing emissions from developed countries (e.g., green-house gases and long range transport of persistent pollutants) and to increasing anthropogenic pressures from the rapid transformation and intensification of their local agriculture and industries. Our current scientific knowledge in ecotoxicology is based mostly on research from temperate systems, especially from Europe and North America. Thus, it is essential that the scientific community addresses current gaps in knowledge regarding the vulnerability of tropical ecosystems to environmental pollution. We need to better understand the environmental distribution of pollutants in tropical systems, in relation to climatic factors such as rainfall, soil type, temperature, and climate change.

Tropical ecosystems such as tropical forests, wetlands, mangroves, and coral reefs host a high biodiversity and provide numerous important ecosystem services to humans, such as fish and food production, and are thus considered very valuable. Some of these tropical ecosystems have been shown to be quite sensitive to pollutants (Amid et al. 2017; Castillo et al. 2000; Hoegh-Guldberg et al. 2007). Tropical ecosystems have often a higher biodiversity than in temperate regions and tropical species are often considered more sensitive than their temperate counterparts due to differences in genetic composition and metabolism (e.g., Freitas and Rocha 2012; Moreira et al. 2014).

An ongoing debate is to what extent toxicological benchmarks and environmental criteria developed for species living in temperate regions are valid and applicable for ecological risk assessment of species in other geographical areas such as in tropical regions (e.g. Daam and Van den Brink 2010; Kwok et al. 2007; Rico et al. 2010). We need to obtain more ecotoxicological data under tropical conditions, using native indigenous test organisms. Several papers in this Special Issue add further knowledge on the sensitivity of tropical species (Arias-Andrés et al. 2016; Daam and Rico 2017; Fournier et al. 2016; Khatikarn et al. 2016; Mansano et al. 2016; Moura et al. 2017; Méndez et al. 2016; Sobrino-Figueroa 2016). Mansano et al. (2016) also discuss the risk of accidently introducing temperate exotic species in tropical ecosystems when using temperate species for toxicity tests. Several authors (e.g. Daam and Van den Brink 2010) have stressed that management of agricultural practices and enforcement of environmental policies are often weak or poorly implemented in tropical countries, leading to a higher pesticide use and environmental contamination than in more developed countries in temperate regions.

In this Special Issue, four papers are presenting ecotoxicological studies in Brazil. Lopes Alves et al. 2016 showed that both the bioavailability and the toxicity of arsenic to soil invertebrates were higher in a tropical soil than in a temperate one, highlighting the importance of using tropical soils from humid regions for deriving Brazilian soil criteria protection values. In the paper of Rietzler et al. 2016, Brazilian researchers conducted a full toxicity identification and evaluation procedure of three eutrophic water reservoirs in Brazil and showed a relationship between eutrophication and metal and phosphate release from the sediment, in turn boosting cyanobacteria blooms in the reservoirs and causing toxicity to water organisms. They suggest that as eutrophication goes faster in tropical regions, climate change may intensify the release of toxic compounds and nutrients from polluted sediments and further accelerate the deterioration of water quality in tropical waters.

In Moura et al. 2017, Brazilian researchers studied the toxicity of the herbicide ametryn, used in tropical sugarcane crops, and found that it affected the embryonic development in *zebra* fish at concentrations found in the environment. Mansano et al. 2016 reported that the native neotropical cladoceran *Ceriodaphnia silvestrii* was more sensitive to the pesticides diuron and carbofuran compared to cladocerans commonly used in temperate regions (*Ceriodaphnia dubia*, *Daphnia magna*, *Daphnia pulex*). Similar results were reported in the paper by Sobrino-Figueroa 2016, where commercial detergents in Mexican waters were found to be more toxic to tropical species (cladocerans, amphipods, and ostracods) than to the temperate cladoceran *Daphnia magna*, commonly used to assess water quality. On the other hand, Khatikarn et al. 2016, found that the antimicrobial compound triclosan was not more toxic to tropical invertebrates from Thailand compared to temperate species using species sensitivity distribution (SSD), supporting that SSDs based on temperate species may be used for risk assessments of this chemical in Thailand.

In the paper by Daam and Rico 2017, the toxicity of tropical and temperate freshwater shrimps to various pesticides was compared. They showed that freshwater shrimps (*Macrobrachium* and *Cardina* sp.) were more sensitive to pesticides than temperate crustaceans such as *Daphnia magna*. They discuss possible reasons for these differences and recommend using native species for first tier prospective ecological risk assessment (ERA).

In the paper by Aguirre-Rubí et al. 2017, the Caribbean oyster *Crassostrea rhizophorae* was evaluated for chemical biomonitoring. The authors found that this oyster, commonly associated with mangrove trees, is a suitable species for biomonitoring in Caribbean coastal systems. Six papers are dealing with pesticide environmental contamination in Costa Rica. Costa Rica has one of the richest biodiversity on Earth. It also has an extensive agriculture, with banana and pineapple as main export crops, and has the highest pesticide use per capita in Central America. Pesticide spray drift and runoff from plantations pose toxicity risks to aquatic organisms downstream these crops and to humans. The paper of Méndez et al. 2016 investigates the toxicity of the fungicide chlorothalonil to three native tadpole species and discusses whether the observed decline of amphibians in Costa Rica can be caused by pesticide application. Fournier et al. 2016 present a toxicity and ecological risk assessment of pesticides in the *Caňo Negro* wetland and watershed located in northern Costa Rica. Arias-Andrés et al. 2016; Echeverría-Sáenz et al. 2016; Rämö et al. 2016 and Svensson et al. 2017 present ecological risk assessments of watersheds located on the Caribbean coast of Costa Rica, polluted from large banana and pineapple plantations, using a suite of laboratory measurements (pesticide residue analyses, toxicity tests) and investigations in situ (biomarkers, benthic invertebrate community analyses) and various risk assessment model approaches to estimate the toxicity of single pesticide compounds and of pesticide mixtures as well (Rämö et al. 2016). Together, these studies show that the pesticide contamination in Costa Rican watersheds is extensive and that mitigation measures are urgently needed to protect Costa Ricas’ biodiversity-rich ecosystems.

Three papers present studies from pesticide toxicity in Vietnam. Tam et al. 2016 show that organophosphate pesticides used in rice cultures in the Mekong Delta may pose a toxicity risk to fish in the rice-pond irrigation system. Stadlinger et al. 2016 compared the pesticide use by rice-fish farmers versus farms that cultivate rice only and pesticides used by farmers that have received training in integrated pest management (IPM) versus non trained farmers (non IPM). They show that rice-fish farmers and farmers that have received IPM training usually choose less toxic pesticides and suggest that further future IPM training is important in Vietnam. In the paper by Amid et al. 2017, the combined effects of the herbicide glyphosate and elevated water temperature were studied on coral bleaching in the Bay of Nha Trang, Vietnam. The effects of herbicide and temperature were found to be additive on coral bleaching. They also found that corals collected from pristine areas were more tolerant than corals collected from polluted sites in Nha Trang Bay. The difference in sensitivity of the corals collected could be explained by different zooxanthellae genotypes.

A paper by Teklu et al. 2016 assessed pesticide toxicity in Ethiopian lakes for human health through drinking water and aquatic organisms. They found a possible risk to aquatic organisms from two pesticides endosulfan and spiroxamine, but no risk to humans. Finally, Mirzaei Aminiyan et al. 2017 assessed heavy metal contamination in road dust in urban environment in the city of Rafsanjan, Iran. The authors showed that the metal concentrations in road dust in Rafsanjan are very high and that the metals As, Cd, Cu, Cr, Pb, and Zn pose a toxicity risk to human health and to the environment.

This Special Issue presents a unique collection of papers, describing various actual ecotoxicological issues in ten tropical and sub-tropical countries. As anthropogenic pressures and environmental problems that these and other tropical countries are currently facing will increase during the coming decades, it is essential that the scientific community carries out more research in tropical regions. We hope that the results and methodologies presented here will help policymakers to motivate stronger environmental protection measures to limit contaminant emissions and habitat destruction in tropical regions.

Finally, we would like to thank all the reviewers who helped improve the quality of the research articles included in this Special Issue.

## References

[CR1] Aguirre-Rubí JR, Luna-Acosta A, Etxebarría N, Soto M, Espinoza F, Ahrens MJ, Marigómez I, (2017) Chemical contamination assessment in mangroved-lined Caribbean coastal systems using the oyster *Crassostrea rhizophorae* as biomonitor species. Environ Sci Pollut Res. 10.1007/s11356-017-9159-2 10.1007/s11356-017-9159-2 28537030

[CR2] Amid C, Olstedt M, Gunnarsson JS, Le Lan H, Tran thi Minh H, Van den Brink PJ, Helström M, Tedengren M (2017) Additive effects of the herbicide glyphosate and elevated temperature on the branched coral *Acropora Formosa* in Nha Trang, Vietnam. Environ Sci Pollut Res. 10.1007/s11356-016-8320-7 10.1007/s11356-016-8320-7 PMC5978828 28111719

[CR3] Arias-Andrés M, Rämö R, Mena Torres F, Ugalde R, Grandas L, Ruepert C, Castillo LE, Van den Brink PJ, Gunnarsson JS (2016) Lower tier toxicity risk assessment of agriculture pesticides detected on the Río Madre de Dios watershed, Costa Rica Environ Sci Pollut Res. 10.1007/s11356-016-7875-7 10.1007/s11356-016-7875-7 27783250

[CR4] Castillo LE, Ruepert C, Solis E (2000). Pesticide residues in the aquatic environment of banana plantation areas in the North Atlantic zone of Costa Rica. Environ Toxicol Chem.

[CR5] Daam MA, Rico A, 2017. Freshwater shrimps as sensitive test species for the risk assessment of pesticides in the tropics. Environ Sci Pollut Res. 10.1007/s11356-016-7451-1 10.1007/s11356-016-7451-1 27530199

[CR6] Daam MA, Van den Brink PJ (2010). Implications of differences between temperate and tropical freshwater ecosystems for the ecological risk assessment of pesticides. Ecotoxicology.

[CR7] Echeverría-Sáenz S, Mena F, Arias-Andrés M, Vargas S, Ruepert C, Van den Brink PJ, Castillo LE, Gunnarsson JS (2016) *In situ* toxicity and ecological risk assessment of agro-pesticide runoff in the Madre de Dios River in Costa Rica. Environ Sci Pollut Res. 10.1007/s11356-016-7817-4 10.1007/s11356-016-7817-4 27757743

[CR8] Fournier ML, Echeverría-Sáenz S, Mena F, Arias-Andrés, de la Cruz E, Ruepert C (2016) Risk assessment of agriculture impact on the Frío River watershed and Caño Negro Ramsar wetland, Costa Rica. Environ Sci Pollut Res. 10.1007/s11356-016-8353-y 10.1007/s11356-016-8353-y 28074363

[CR9] Freitas EC, Rocha O (2012). Acute and chronic effects of atrazine and sodium dodecyl sulfate on the tropical freshwater cladoceran *Pseudosida ramosa*. Ecotoxicology.

[CR10] Hoegh-Guldberg MPJ, Hooten AJ, Steneck SR, Greenfield P, Gomez E, Harvell CD, Sale PF, Edwards AJ, Caldeira K, Knowlton N, Eakin CM, Iglesias-Prieto R, Muthiga N, Bradbury RH, Dubi A, Hatziolos ME (2007). Coral reefs under rapid climate change and ocean acidification. Science.

[CR11] Khatikarn J, Satapornvanit K, Price OR, Van den Brink PJ (2016) Effects of triclosan on aquatic invertebrates in tropics and the influence of pH on its toxicity on microalgae. Environ Sci Pollut Res. 10.1007/s11356-016-7302-0 10.1007/s11356-016-7302-0 PMC5978822 27543130

[CR12] Kwok KWH, Leung KMY, Chu VKH, Lam PKS, Morritt D, Maltby L, Brock TCM, Van den Brink PJ, Warne MSJ, Crane M (2007). Comparison of tropical and temperate fresh-water animal species’ acute sensitivities to chemicals: implications for deriving safe extrapolation factors. Integr Environ Assess Manag.

[CR13] Lopes Alves PR, Barbosa da Silva E, Nogueira Cardoso EJB, Ferracciú Alleoni LR (2016) Ecotoxicological impact of arsenic on earthworms and collembolans as affected by attributes of a highly weathered tropical soil. Environ Sci Pollut Res. 10.1007/s11356-016-6839-2 10.1007/s11356-016-6839-2 27178288

[CR14] Mansano AS, Moreira RA, Dornfeld HC, Diniz LGR, Vieira EM, Daam MA, Rocha O, Seleghim MHR (2016) Acute and chronic toxicity of diuron and carbofuran to the neotropical cladoceran *Ceriodaphnia silvestrii*. Environ Sci Pollut Res. 10.1007/s11356-016-8274-9 10.1007/s11356-016-8274-9 28004367

[CR15] Méndez M, Obando P, Pinnock-Branford M, Ruepert C, Castillo LE, Mena F, Alvarado G (2016). Acute, chronic and biochemical effects of chlorothalonil on *Agalychnis callidryas* and tadpoles. Environ Sci Pollut Res.

[CR16] Mirzaei Aminiyan M, Baalousha M, Mousavi R, Mirzaei Aminiyan F, Hosseini H, Heydariyan A (2017) The ecological risk, source identification, and pollution assessment of heavy metals in road dust: a case study in Rafsanjan, SE Iran Environ Sci Pollut Res. 10.1007/s11356-017-8539-y 10.1007/s11356-017-8539-y 28255819

[CR17] Moreira RA, Mansano AS, Silva LCD, Rocha O (2014). A comparative study of the acute toxicity of the herbicide atrazine to cladocerans *Daphnia magna*, *Ceriodaphnia silvestrii* and *Macrothrix flabelligera*. Acta Limnol Bras.

[CR18] Moura MAM, Oliveira R, Jonsson CM, Domingues I, Soares AMVM, Nogueira AJA, 2017. The sugarcane herbicide ametryn induces oxidative stress and developmental abnormalities in zebrafish embryos. Environ Sci Pollut Res. 10.1007/s11356-017-9614-0 10.1007/s11356-017-9614-0 28702912

[CR19] Rämö RA, Van den Brink PJ, Ruepert C, Castillo LE, Gunnarsson JS (2016) Environmental risk assessment of pesticides in the River Madre de Dios, Costa Rica using PERPEST, SSD, and msPAF models. Environ Sci Pollut Res. 10.1007/s11356-016-7375-9 10.1007/s11356-016-7375-9 PMC5978829 27617335

[CR20] Rico A, Geber-Corrêa R, Campos PS, Garcia MVB, Waichman AV, Van den Brink PJ (2010). Effect of parathion-methyl on Amazonian fish and freshwater invertebrates: a comparison of sensitivity with temperate data. Arch Environ Contam Toxicol.

[CR21] Rietzler AC, Botta CR, Ribeiro MM, Rocha O, Fonseca AL (2016) Accelerated eutrophication and toxicity in tropical reservoir water and sediments: an ecotoxicological approach. Environ Sci Pollut Res. 10.1007/s11356-016-7719-5 10.1007/s11356-016-7719-5 27761862

[CR22] Ripple WJ (2017). World scientists’ warning to humanity: a second notice. Bioscience.

[CR23] Sobrino-Figueroa A (2016) Toxic effect of commercial detergents on organisms from different trophic levels. Environ Sci Pollut Res. 10.1007/s11356-016-7861-0 10.1007/s11356-016-7861-0 27757746

[CR24] Stadlinger N, Berg H, Van den Brink PJ, Tam NT, Gunnarsson JS (2016) Comparison of predicted aquatic risks of pesticides used under different rice-farming strategies in the Mekong Delta, Vietnam Envir Sci Pollut Res. 10.1007/s11356-016-7991-4 10.1007/s11356-016-7991-4 PMC5978820 27854060

[CR25] Svensson O, Sanderson Bellamy A, Van den Brink PJ, Tedengren M, Gunnarsson JS (2017) Assessing the ecological impact of banana farms on water quality using aquatic macroinvertebrate community composition. Environ Sci Pollut Res. 10.1007/s11356-016-8248-y 10.1007/s11356-016-8248-y PMC5978817 28116625

[CR26] Tam NT, Berg H, Cong NV (2016) Evaluation of the joint toxicity of chlorpyrifos ethyl and fenobucarb on climbing perch (*Anabas testudineus*) from rice fields in the Mekong Delta, Vietnam Environ Sci Pollut Res. 10.1007/s11356-016-6980-y 10.1007/s11356-016-6980-y 27250094

[CR27] Teklu BM, Hailu A, Wiegant DA, Scholten BS, van den Brink PJ (2016) Impacts of nutrients and pesticides from small- and large-scale agriculture on the water quality of Lake Ziway, Ethiopia Environ Sci Pollut. 10.1007/s11356-016-6714-1 10.1007/s11356-016-6714-1 PMC5978843 27126865

[CR28] UN (2017) United Nations, Department of Economic and Social Affairs, Population Division. World population prospects: the 2017 revision, key findings and advance tables. Working paper no. ESA/P/WP/248

